# Metformin improves skeletal muscle microvascular insulin resistance in metabolic syndrome

**DOI:** 10.1152/ajpendo.00287.2021

**Published:** 2021-12-27

**Authors:** Linda A. Jahn, Lee Hartline, Zhenqi Liu, Eugene J. Barrett

**Affiliations:** ^1^Division of Endocrinology, Department of Medicine, University of Virginia, School of Medicine, Charlottesville, Virginia; ^2^Department of Pharmacology, University of Virginia, School of Medicine, Charlottesville, Virginia

**Keywords:** contrast-enhanced ultrasound, insulin resistance, metformin, microvasculature

## Abstract

Microvascular insulin resistance is present in metabolic syndrome and may contribute to increased cardiovascular disease risk and the impaired metabolic response to insulin observed. Metformin improves metabolic insulin resistance in humans. Its effects on macro and microvascular insulin resistance have not been defined. Eleven subjects with nondiabetic metabolic syndrome were studied four times (before and after 12 wk of treatment with placebo or metformin) using a crossover design, with an 8-wk washout interval between treatments. On each occasion, we measured three indices of large artery function [pulse wave velocity (PWV), radial pulse wave separation analysis (PWSA), brachial artery endothelial function (flow-mediated dilation-FMD)] as well as muscle microvascular perfusion [contrast-enhanced ultrasound (CEU)] before and at 120 min into a 150 min, 1 mU/min/kg euglycemic insulin clamp. Metformin decreased body mass index (BMI), fat weight, and % body fat (*P* < 0.05, each), however, placebo had no effect. Metformin (not placebo) improved metabolic insulin sensitivity, (clamp glucose infusion rate, *P* < 0.01), PWV, and FMD after insulin were unaffected by metformin treatment. PWSA improved with insulin only after metformin *P* < 0.01). Insulin decreased muscle microvascular blood volume measured by contrast ultrasound both before and after placebo and before metformin (*P* < 0.02 for each) but not after metformin. Short-term metformin treatment improves both metabolic and muscle microvascular response to insulin. Metformin’s effect on microvascular insulin responsiveness may contribute to its beneficial metabolic effects. Metformin did not improve aortic stiffness or brachial artery endothelial function, but enhanced radial pulse wave properties consistent with relaxation of smaller arterioles.

**NEW & NOTEWORTHY** Metformin, a first-line treatment for type 2 diabetes, is often used in patients with insulin resistance and metabolic syndrome. Here, we provide the first evidence for metformin improving muscle microvascular insulin sensitivity in insulin-resistant humans. Simultaneously, metformin improved muscle glucose disposal, supporting a close relationship between insulin’s microvascular and its metabolic actions in muscle. Whether enhanced microvascular insulin sensitivity contributes to metformin’s ability to decrease microvascular complications in diabetes remains to be resolved.

## Graphical abstract

**Figure:**
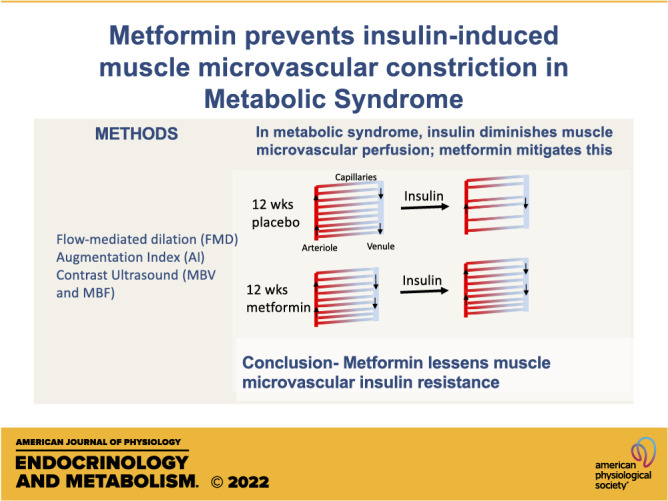

## INTRODUCTION

Metabolic syndrome closely associates with metabolic insulin resistance and increases the cardiovascular disease (CVD) event risk (1). Clinical interventions to lessen the risk of adverse outcomes from metabolic syndrome consist of lifestyle modification and treating syndrome components (i.e., hypertension, obesity, dysglycemia, hypertriglyceridemia, and low HDL cholesterol) (2). Other than dysglycemia, metformin does not target specific metabolic syndrome components. Metformin’s effect on CVD clinical outcomes is uncertain. A beneficial effect was seen in UKPDS (3) for patients with obesity and type 2 diabetes, but other studies have been less convincing and a recent meta-analysis underscores metformin’s uncertain CVD effect (4).

Considering markers of vascular health, in one study of subjects with type 2 diabetes (DM2), metformin had a beneficial effect on carotid intima-media thickness (cIMT) (a vascular surrogate for arterial health) (5). However, another study of patients with coronary disease, but no diabetes, reported no significant effect on cIMT (6) and another study yielded negative cIMT results in patients with type 1 diabetes (7).

Endothelial dysfunction (8) and vascular stiffness (9) each independently predict adverse CVD outcomes and each is reported impaired in metabolic syndrome (10, 11) and other insulin-resistant states (12). Vascular stiffness particularly associates with hypertension as a metabolic syndrome component (13).

Insulin has acute vasodilatory effects (14–16) in health, which depend on its enhancing endothelial nitric oxide (NO) production (17, 18). We recently reported that insulin acutely (hyperinsulinemic-euglycemic clamp) augments brachial artery flow-mediated dilation (FMD) and expands muscle microvascular blood volume (MBV) in healthy middle-aged volunteers (19). However, both these actions were impaired in age-matched patients with nondiabetic metabolic syndrome (19). This led us to conclude that metabolic syndrome was characterized by both macro and microvascular insulin resistance due, at least in part, to a diminished nitric oxide-mediated vasodilatory action of insulin. Baseline vascular stiffness was greater in the metabolic syndrome group (19), [measured either as pulse wave velocity (PWV) or augmentation index (AI)]. In subjects with metabolic syndrome only, AI declined while PWV increased with insulin infusion. The role of NO in either of these measures is uncertain.

Metformin improves metabolic insulin resistance in a variety of clinical settings. For insulin-resistant patients with metabolic syndrome, metformin was reported to improve baseline (postabsorptive) endothelial function in two studies (20, 21) but not in another (22). Metformin treatment of subjects with insulin resistance and polycystic ovarian syndrome (23) may improve postabsorptive vascular stiffness.

The current study was undertaken to specifically test in humans whether metformin treatment of metabolic syndrome affected either basal macro or microvascular function or responses to acute hyperinsulinemia, which, to our knowledge, has not been tested previously.

## METHODS

### Human Subjects

Studies were performed on 12 subjects (7 F/5M) meeting criteria for metabolic syndrome as defined by AHA-NHLBI guidelines. Subjects were middle-aged (52 ± 1.7 yr), nonsmokers. Five subjects were on stable doses of antihypertensive medications at the time of recruitment and four were on statins. Subjects were not taking any other medications or supplements known to affect vascular function (e.g., angiotensin-converting enzyme inhibitors, angiotensin or adrenergic receptor blockers, fish oil, vitamins E or C, or aspirin) and none were diabetic. The study protocol was approved by the University of Virginia Institutional Review Board. All studies were performed in the University of Virginia Clinical Research Unit (CRU).

### Experimental Protocol

**Figure 1.**
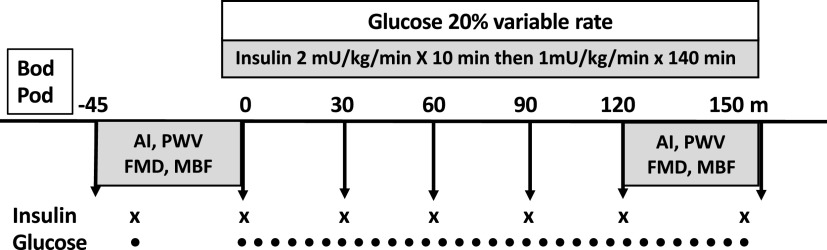
Within 2 wk of screening and randomization, volunteers were admitted to the clinical research unit (CRU) after fasting from 10 PM, the previous evening for the first of four outpatient admissions, where metabolic, body composition (Bod Pod), and vascular measures were obtained. These consisted of fasting and end of the study blood draws as well as insulin measurements every 30 min and glucose every 5 min during the euglycemic-hyperinsulinemic clamp. Vascular studies began with pulse wave analysis and PWV followed by FMD and MBV. The outpatient admission (metabolic and vascular measures) was repeated after the first 12-wk treatment and again before and after the second 12-wk treatment (with 8-wk washout in between). AI, augmentation index; FMD, flow-mediated dilation; MBF, microvascular blood flow; MBV, microvascular blood volume; PWV, pulse wave velocity.

Subjects were recruited by newspaper advertisement, followed by telephone interviews to review inclusion/exclusion criteria. Subsequently, each gave written informed consent at a screening visit, which included a medical history and physical exam, measurement of serum electrolytes, liver and renal function, a complete blood count, fasting glucose and lipid profile, and a pregnancy test (if applicable). Subjects meeting screening criteria were randomized to receive 12 wk of either placebo or metformin treatment. This was followed by an 8-wk “washout” interval of study drugs followed by crossover to the alternate treatment. Eight weeks was four to eight times longer than the time allowed for metformin washout in the Diabetes Prevention Program (24). To minimize gastrointestinal side effects, both metformin and placebo tablets (500 mg) were initiated at a dose of 1 tablet/day for 1 wk, then 2 tabs (breakfast and dinner) for week 2, and then 3 tabs/day for *weeks 3–12*. One female subject developed significant gastrointestinal (GI) side effects on metformin and withdrew after only baseline studies were obtained. All others completed both 12 wk drug regimens and all studies. The history, physical, and laboratory tests performed at screening were repeated before each outpatient admission. Measurements of vascular function were made both in the fasting state and between 120 and 150 min of insulin infusion before and after each 12-wk treatment protocol (Fig. 1). We measured body composition using the Bod-Pod air displacement method as previously described before and after each 12-wk treatment interval (19). For measurements of metabolic and vascular function, subjects were admitted to the Clinical Research Unit (CRU) at 7 AM after fasting from 10 PM the evening before. Body composition measurements were obtained followed by baseline samples for glucose and insulin and measurements of PWSA, PWV, FMD, and microvascular blood volume, and microvascular flow velocity (CEU) (Fig. 1). After these measurements were completed, a 150 min euglycemic-hyperinsulinemic clamp was begun and vascular measurements were repeated between 120 and 150 min of the clamp. Glucose was measured every 5 min and insulin every 30 min during the clamp.

### Measures of Conduit Vessel Function

Vascular stiffness was assessed by performing both pulse wave separation analysis and measurement of carotid-femoral pulse wave velocity (cfPWV) measured using applanation tonometry (SphygmoCor system, AtCor Medical, Itasca, IL). For pulse wave analysis, we used the radial artery and the measured AI (corrected to a heart rate of 75 beats/min), wave separation analysis including measurement of forward (Pf) and backward (Pb) pulse wave amplitude as well as reflection magnitude (RM = Pb/Pf) (25, 26). PWV was measured between the carotid and femoral artery. FMD was determined using a Sequoia C512 (Siemens-Acuson, Mountain View, CA). The brachial artery was imaged ∼5 cm proximal to the antecubital crease using B-mode ultrasound. Baseline brachial artery diameter was measured and a forearm blood pressure cuff was inflated to 250 mmHg for 5 min then deflated. Diameter was obtained every 10 s from 30 to 120 s post cuff deflation. Analysis for vessel diameter was done offline using Brachial Analyzer (Medical Imaging Applications, LLC, Coralville, IA).

### Measurement of Skeletal Muscle Microvascular Perfusion

Contrast-enhanced ultrasound **(**CEU) of forearm skeletal muscle was performed using a Sequoia C512, a linear transducer 15L8W (Siemens-Acuson, Mountain View, CA), and contrast pulse sequencing (CPS) during the continuous systemic infusion of Definity (Lantheus Medical Imaging, North Billerica, MA) as described previously (27). MBV and microvascular flow velocity (MFV) were each measured immediately before and between 120 and 150 min of the insulin clamp and their product, microvascular blood flow (MBF) was calculated.

### Euglycemic Hyperinsulinemic Clamp

After obtaining baseline plasma samples and vascular measurements, a 2 mU/kg/min insulin infusion was started and decreased to 1 mU/kg/min after 10 min and maintained until 150 min. During the insulin clamp to avoid the confounding systemic vasodilatory effect of limb heating (28, 29), we did not use a “heated hand” to arterialize venous blood, as previously described (30).

### Biochemical Analyses

Serum electrolytes, creatinine, liver function test, HbA1c, complete blood count, lipid profile, and pregnancy tests were assayed at the University of Virginia Clinical Chemistry Laboratory. Plasma glucose was measured using a YSI glucose analyzer (Yellow Spring Instruments) and insulin was determined using a Siemens Healthcare Diagnostics Immulite 2000 random access analyzer in the CRU core laboratory.

### Statistics

The primary endpoint for microvascular insulin responsiveness was the change in forearm muscle MBV from baseline to end-of-clamp. Secondary endpoints were arterial stiffness (cfPWV, AI, Pf, Pb, and RM), FMD and the glucose infusion rate (GIR) needed to maintain euglycemia. Data are presented as the means ± standard error of the mean (SEM). Comparisons were made within treatment (pre vs. post) and between treatments at baseline and at 150 min when the insulin clamp was finished using two-way repeated-measures ANOVA or paired Student’s *t* test where appropriate and Pearson’s correlation using SigmaStat 3.2 (Systat Software, San Jose, CA). Statistical significance was considered when *P* ≤ 0.05 for primary and secondary endpoints.

## RESULTS

### Subject Characteristics

Eleven subjects (6 F/5M), age 52 ± 1.7 yr completed the 12 wk of metformin and 12 wk of placebo treatment in a randomized order. Six received metformin first and five received placebo first. As shown in Table 1, the 12 wk of placebo treatment did not affect BMI, fasting plasma glucose, systolic or diastolic blood pressure, lipid values, percent body fat, or waist circumference. In contrast, BMI, fat weight, and percent body fat declined with metformin treatment (Table 1). Lean body mass and fasting plasma glucose did not change with either metformin or placebo and plasma lipids were likewise unaffected. Compared with either pretreatment or placebo treatment, fasting plasma insulin was lower after 12 wk of metformin (*P* < 0.01), suggesting increased insulin sensitivity. Fasting plasma glucose was lower (*P* < 0.05), as was BMI (*P* < 0.05), fat weight (*P* < 0.01), and percent body fat (*P* < 0.01) in subjects before receiving placebo compared with before receiving metformin. This may have been secondary to a “carryover” effect of prior metformin treatment in the randomization procedure that was not reversed by the 8 wk washout. Metformin does not appear to affect insulin clearance, so the lower insulin concentration likely reflects improved sensitivity (31).

**Table 1.** Subject characteristics

	Preplacebo	Postplacebo	Premetformin	Postmetformin
BMI, kg/m^2^	36.5 ± 2.1	36.2 ± 2.1	37.5 ± 2.2	36.2 ± 2.1*
Fasting glucose, mg/dL	97.6 ± 2.0	99.6 ± 2.5	103.5 ± 2.7	98.2 ± 2.4
Systolic BP, mmHg	125.6 ± 4.4	124.1 ± 4.3	131.2 ± 5.1	127.7 + 3.8
Diastolic BP, mmHg	70.7 ± 3.7	72.4 ± 3.1	78.6 ± 3.0	74.0 + 3.3
Cholesterol, mg/dL	218 ± 26	225.5 ± 35	195.2 ± 9.1	184.6 ± 12.2
LDL-cholesterol, mg/dL	129 ± 8	126 ± 5	122.4 ± 7.2	115.7 ± 9.2
HDL-cholesterol, mg/dL	45 ± 4	42.1 ± 3.4	43.6 ± 4.1	43.5 ± 4.9
Triglycerides, mg/dL	166 ± 46	215 ± 84	176.2 ± 23.3	147.3 ± 12.2
Lean weight, kg	60.9 ± 3.5	59.7 ± 3.4	60.45 ± 3.4	59.9 ± 3.4
Fat weight, kg	45.7 ± 4.4	45.3 ± 4.3	47.6 ± 4.6	45.3 ± 4.7*
% Fat	42.5 ± 2.7	42 ± 3.1	43.6 ± 2.81	42.4 ± 3.1*
Fasting insulin, pM	77 ± 18	75 ± 17	73 ± 12	50 ± 8*
Waist circumference, cm	112.6 ± 2.8	110.9 ± 2.7	114.5 ± 2.8	113.3 ± 2.3

No significant change in any variable was seen when comparing before and after placebo. *P < 0.01 for the comparison before and after metformin. BMI, body mass index.

Considering measures of macro and microvascular function, as shown in Table 2, there were no significant within-group differences in baseline measures of cfPWV, FMD, AI, Pf, Pb, or RM before and after treatment with placebo or metformin, except for a lower baseline RM after metformin. In addition, there were no between-group differences in baseline values for any of these variables between the placebo and metformin-treated groups either before or after treatment (Table 2).

**Table 2.** Measures of vascular function

	Preplacebo		Postplacebo		Premetformin		Postmetformin	
Insulin	−	+	−	+	−	+	−	+
PWV, M/s	7.6 ± 0.5	7.8 ± 0.5	7.5 ± 0.3	8.0 ± 0.6	7.4 ± 0.3	7.9 ± 0.7	7.5 ± 0.3	7.5 ± 0.3
FMD, %	5.4 ± 1	5.3 ± 1.4	6.9 ± 1.5	6.1 ± 1.1	5.3 ± 0.8	4.7 ± 1.1	5.9 ± 1.1	6.2 ± 1.1
AI, %	22 ± 3	17 ± 4	20 ± 4	20 ± 5	21 ± 3	18 ± 4	19 ± 4	16 ± 4
Forward wave, Pf	30 ± 3	30 ± 2	31 ± 3	30 ± 2	30 ± 3	28 ± 2	33 ± 2	30 ± 2
Reflectedwave, Pb	21 ± 1	20 ± 2	20 ± 2	19 ± 2	20 ± 2	18 ± 2	19 ± 2	16 ± 1*
Reflection magnitude, RM	73 ± 3	62 ± 7	68 ± 7	67 ± 8	67 ± 6	64 ± 6	61 ± 5#	56 ± 6#
Preischemic brachial artery diameter, mm	4.6 ± 0.4	4.5 ± 0.3	4.4 ± 0.3	4.5 ± 0.3	4.4 ± 0.4	4.4 ± 0.3	4.3 ± 0.3	4.5 ± 0.3*
Postischemic brachial artery diameter, mm	4.8 ± 0.4	4.8 ± 0.3	4.7 ± 0.3	4.8 ± 0.3	4.6 ± 0.3	4.5 ± 0.3	4.5 ± 0.3	4.7 ± 0.3*

*P < 0.01 insulin effect postmetformin, #*P* < 0.05 pre- vs. postmetformin with or without insulin. AI, augmentation index; FMD, flow-mediated dilation; PWV, pulse-wave velocity.

Similarly, in muscle microvasculature, there were no significant within-group changes of baseline MBV following 12 wk of either placebo or metformin (1.85 ± 0.4 vs. 1.82 ± 0.3, and 1.30 ± 0.1 vs. 1.94 ± 0.4 video intensity units, respectively). Furthermore, there were no between-group differences in baseline microvascular blood flow either before or after each 12-wk treatment interval.

### Metabolic Insulin Response

**Figure 2.**
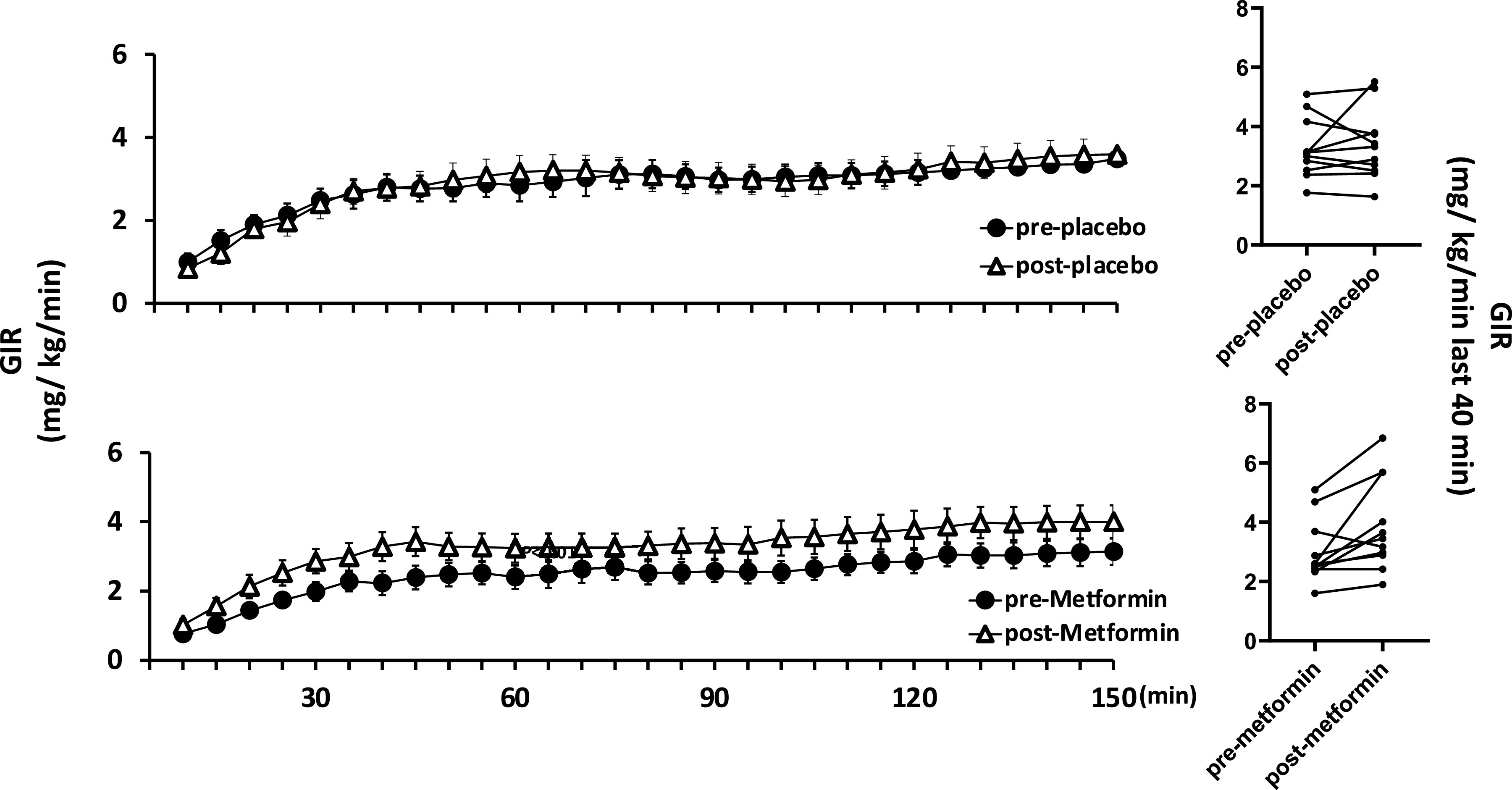
The time course of the glucose infusion rate (GIR) measured during the insulin clamp is shown before and after placebo (*top*) and before and after metformin (*bottom*). The right most two panels show the individual data for the steady-state (last 40 min) GIR before and after placebo (*top*) and before and after metformin (*bottom*).

The glucose infusion rate throughout the insulin clamp (Fig. 2) was not affected by placebo treatment (3.3 ± 0.3 vs. 3.4 ± 0.4 mg/min/kg at a steady state during the last 40 min of the euglycemic clamp). Twelve weeks of metformin treatment, by contrast, increased steady-state GIR (3.0 ± 0.3 vs. 3.9 ± 0.5 mg/min/kg, *P* < 0.01). Plasma insulin concentrations both fasting (51 ± 7 and 75 ± 15 pM) and at steady state (150 min) during the clamp (445 ± 43 vs. 565 ± 64 pM) were lower after 12 wk of metformin than pretreatment, *P* < 0.02) and plasma glucose concentrations were not different between groups during the clamp. Consequently, the enhanced glucose disposal during metformin treatment appeared secondary to increased metabolic insulin sensitivity.

### Vascular Insulin Responses

Considering first vascular stiffness as measured by cfPWV and pulse wave analysis, we were able to measure cfPWV in only seven of 11 subjects. Thick subcutaneous adipose tissue in four subjects with obesity prevented obtaining their femoral and/or carotid pulse waveforms. In this small sample, there were no apparent cfPWV changes in subjects either following placebo or metformin treatment or in response to insulin. Radial artery pulse wave separation analysis indicated that the Pb declined during insulin infusion only after metformin treatment (Table 2) and the RM was lower both before and during insulin infusion with metformin but not placebo treatment. As shown in Table 2, there were no significant baseline changes in AI in response to metformin or placebo treatment, and no effect of insulin was noted. Thus, 12 wk of metformin or placebo did not appear to influence baseline aortic stiffness or affect aortic stiffness in response to insulin. The changes in Pb and RM suggest a metformin effect to relax smaller arterioles.

Brachial artery endothelial function, as indexed by FMD, was measured pre and posttreatment with metformin or placebo in the fasting state and between 120 and 150 min of insulin. As shown in Table 2, neither placebo nor metformin affected baseline FMD and insulin did not affect FMD at any time. However, we noted that in the metformin-treated subjects the pre- and postischemic brachial artery diameter was significantly increased above baseline after 150 min of insulin (*P* < 0.001 for each). This was not seen either before metformin or pre- or posttreatment in the placebo group (Table 2) and suggests metformin treatment may have improved brachial artery insulin responsiveness.

**Figure 3.**
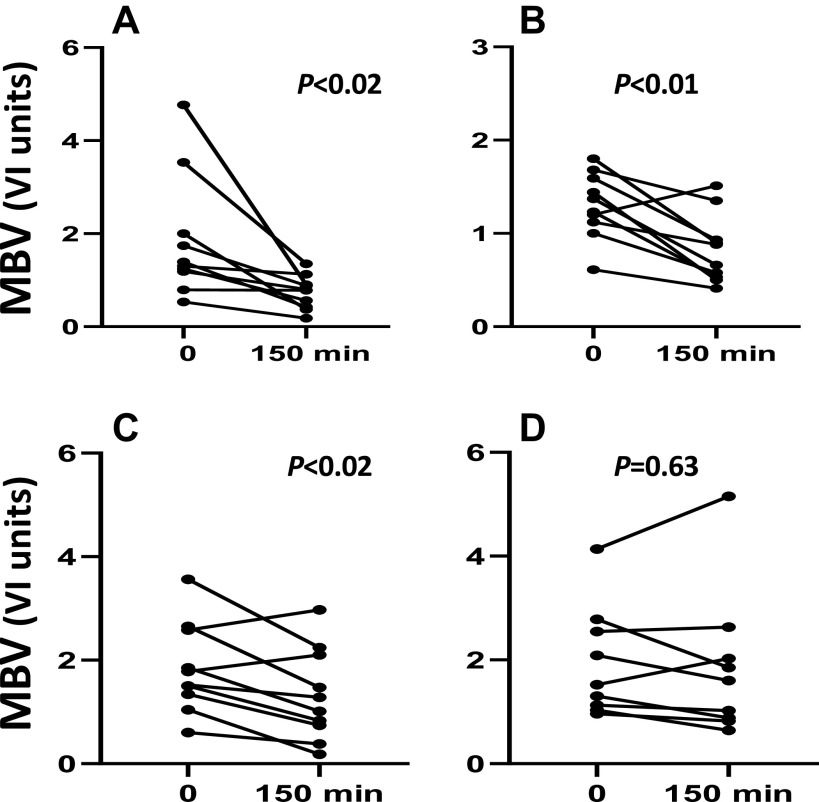
The change in microvascular blood volume (MBV) in response to 150 min of insulin infusion before (*A*) and after 12 wk of placebo (*C*) and before (*B*) and after (*D*) 12 wk of metformin. Units of MBV reflect the video intensity of the ultrasound image. *P* values reflect paired *t* test results for before and after insulin.

Considering skeletal muscle microvasculature, baseline fasting microvascular blood volume (MBV) and microvascular blood flow (MBF) were not different before and after 8 wk of either placebo or metformin. Microvascular endothelial insulin responsiveness was assessed as the change of MBV, microvascular flow velocity (MFV), and their product MBF with insulin infusion before and after either placebo or metformin treatment. When tested before either placebo or metformin treatment, or after placebo treatment, insulin decreased MBV (*P* < 0.02, for each), indicating a “derecruitment” of muscle capillaries secondary to arteriolar vasoconstriction (Fig. 3*, A–C*). This decrease of MBV would lessen endothelial surface area accessed by blood and may impair solute exchange. This contrasts with insulin’s effect to increase MBV in healthy, insulin-sensitive subjects reported previously (19, 30, 32), which could enhance solute exchange. However, metformin treatment for 12 wk dampened the vasoconstrictor response (Fig. 3*D*) to insulin infusion, and the % decline of MBV with insulin after metformin was less than seen either before metformin or before or after placebo treatment (*P* < 0.05, for each comparison).

**Figure 4.**
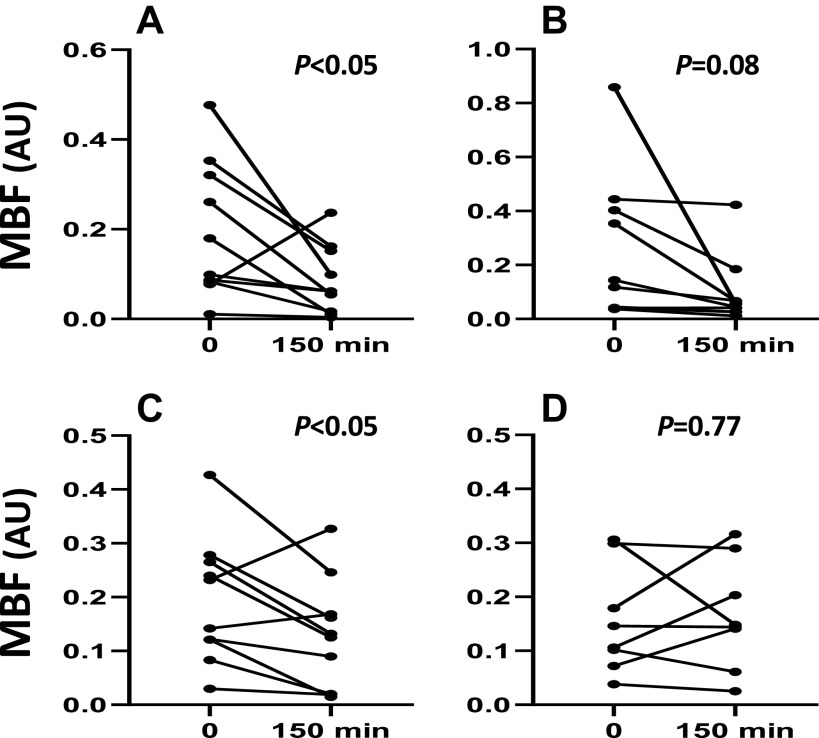
The change in microvascular blood flow (MBF) in response to 150 min of insulin infusion before (*A*) and after 12 wk of placebo (*C*) and before (*B*) and after (*D*) 12 wk of metformin. *P* values reflect paired *t* test results for before and after insulin.

**Figure 5.**
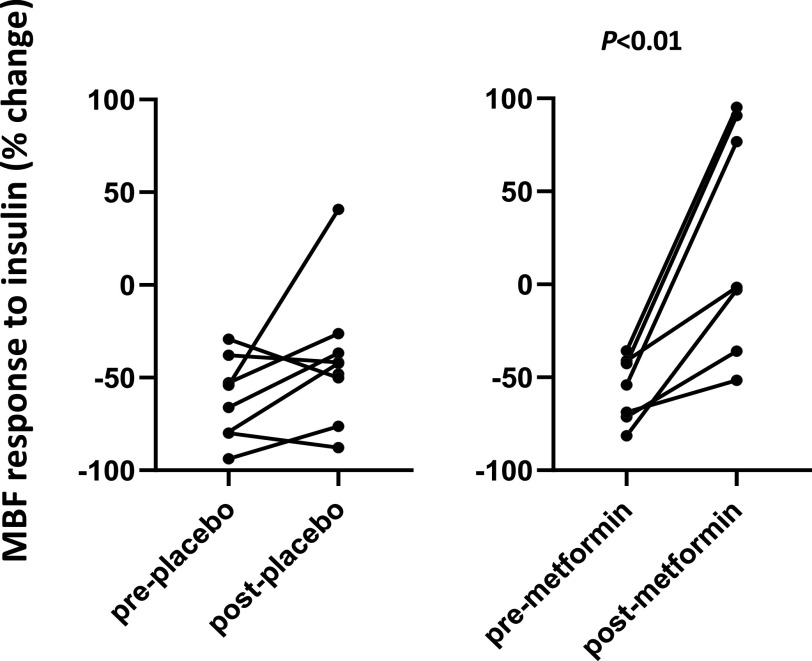
The percent change in MBF in response to 150 min of insulin before and after either placebo (*left*) or metformin (*right*) are shown for the seven individuals in whom good microvascular images were obtained on all four study days. *P* value reflects the comparison pre- vs. postmetformin (paired *t* test). MBF, microvascular blood flow.

Microvascular flow velocity (MFV) did not change significantly with insulin infusion pre or postmetformin or placebo. MBF, the product of MBV × MFV, decreased significantly with insulin infusion both before and after placebo treatment (Fig. 4, *A*and *C*) and trended downward before metformin treatment (*P* = 0.08, Fig. 4*B*), consistent with an insulin-induced perfusion impairment. After metformin, there was no significant change of MBF with insulin treatment. Comparing the muscle MBF response with insulin before and after either placebo or metformin in seven subjects in whom satisfactory images were obtained before and after insulin in all four studies (Fig. 5), there was a significant (*P* < 0.01) effect of metformin on MBF not seen with placebo. Insulin no longer provoked a vasoconstrictive response in muscle microvasculature, metformin treatment appeared to relieve this paradoxical insulin response.

## DISCUSSION

Metformin is the preferred “insulin-sensitizing” drug used to treat DM2. Its mechanism(s) of action remain a topic of investigation (33–36). It is also often considered as an off-label treatment option in patients with prediabetes or metabolic syndrome. In part, the latter consideration is prompted by the increased CVD risk of these patients and reports that metformin may have cardiovascular benefits beyond its actions to lower blood glucose (37, 38) though this is not evident in all trials (39, 40). Short-term studies demonstrate that metformin can diminish vascular stiffness (23), decrease circulating markers of endothelial injury (41), and improve endothelial function in subjects with diabetes or prediabetes (21, 42). Longer-term metformin use in DM2 may improve CVD outcomes (38). Whether metformin’s “insulin-sensitizing” action might extend beyond metabolic insulin resistance to include effects on macro or microvascular insulin resistance, to our knowledge, had not been previously tested in humans.

To our knowledge, the current study provides the first evidence that 12 wk of metformin improves the acute microvascular response to insulin within skeletal muscle. We did not, in this study, measure total forearm muscle blood flow. The MBV, measured here by CEU, provides a measure of endothelial surface exposure. As the Fick principle indicates, solute exchange within an organ is a function of *1*) solute concentration gradient; *2*) blood flow; *3*) endothelial surface area available for solute exchange, and *4*) vessel wall vascular permeability to a given solute. Metformin, by improving the MBV response to insulin can enhance solute exchange independent of total muscle blood flow changes. It is noteworthy that in response to insulin, MBV declined both before and after treatment with either metformin or placebo, but the decline is significantly less after metformin. We have previously observed in both rodents (43) and humans (19) that vascular insulin resistance shifts the balance of insulin’s microvascular action from a net vasodilation (increasing MBV) to a net vasoconstrictive effect with a decline in microvascular perfusion (MBF). This may be due to pathway-selective insulin resistance (44) with inhibition of nitric oxide synthase stimulation but persistent insulin stimulation of endothelin release. Supporting this possibility, in the rat, blocking endothelin action prevents insulin’s vasoconstrictive effect on muscle microvasculature during free fatty acid-induced insulin resistance (43). Endothelin blockade also enhances leg blood flow responses to methacholine in patients with type 2 diabetes and obesity (45). However, this interpretation may be overly simplistic, and the role of pathway selective insulin resistance in the microvasculature needs to be further explored.

As discussed previously (46), improving microvascular perfusion advantages delivery of insulin and glucose to myocytes by increasing the endothelial surface area available for their exchange. Thus, the improved microvascular insulin sensitivity seen here may contribute to the improved metabolic actions of insulin after metformin. We emphasize that while the vascular and metabolic responses to insulin are improved, they are by no means normalized. This is evident from the vasoconstrictive response to insulin still seen in the majority of the metformin-treated subjects and lower steady-state GIR after metformin treatment (3.9 ± 0.5 mg/min/kg between 120 and 150 min of the clamp) in the current study compared with that seen previously after 2 h of an identical insulin infusion in middle-aged, lean healthy adults (5.8 ± 0.4 mg/min/kg) (19). A previous study reported a similar vasoconstrictor response to insulin in persons with type 2 diabetes (47). Treatment with Iloprost improved insulin microvascular responsiveness, without improving metabolic responses to insulin.

Insulin also increased baseline and postischemic brachial artery diameter compared with the fasting values, only in the postmetformin treatment group (Table 2). We did not measure brachial flow velocity in these subjects, but the increase in diameter would be consistent with an effect of metformin to increase forearm flow in response to insulin. As for muscle microvasculature, this effect on a large muscular artery may indicate an effect of metformin to enhance vascular insulin sensitivity.

Interestingly, we did not see any effect of metformin on baseline brachial artery endothelial function measured by FMD. Using methacholine infusion to measure endothelial function, the response to insulin was reported to be impaired by obesity and DM2 (48). Others have reported that 3 mo of metformin markedly increased baseline FMD in subjects with metabolic syndrome (20), whereas in DM2 either improvement (49) or no effect was seen (50). Currently, the basis for these divergent findings is not apparent but may be due to differences in the clinical populations studied. The improvement in radial pulse wave analysis variables after metformin seen here (Table 2) is consistent with relaxation of arteriolar vessels that would diminish the reflected wave amplitude and reflection magnitude. Several studies have suggested that Pb and RM are better predictors of CVD outcomes than AI (25, 39, 51).

We previously have seen that insulin acutely enhances FMD in healthy middle-aged adults but not in subjects with metabolic syndrome (19). Likewise, in subjects with obesity, acute hyperinsulinemia did not enhance FMD (52). It is possible that in the current study the significant increase in postinsulin brachial artery diameter seen after metformin treatment (both before and after the FMD-induced ischemic stress), obscured our ability to detect an acute response to ischemia.

In closing, we confirm that metformin improves metabolic insulin sensitivity in subjects with metabolic syndrome and provide the first evidence that it also improves, without normalizing, muscle microvascular insulin sensitivity. Muscle microvascular responses to insulin, in part, determine insulin’s muscle metabolic action (46). The current findings suggest that metformin’s vascular insulin sensitization may contribute part of metformin’s action to enhance muscle metabolic insulin sensitivity. As these subjects were not significantly hyperglycemic, the effect of metformin is unlikely secondary to improved glucose control.

## GRANTS

This work was supported by research grants from the National Institutes of Health (NIH) [1RO1DK073059 (to E.J.B.) and 1R01DK102359, RO1DK125330, and RO1DK124344 (to Z.L.)].

## DISCLOSURES

No conflicts of interest, financial or otherwise, are declared by the authors.

## AUTHOR CONTRIBUTIONS

E.J.B. conceived and designed research; L.A.J., L.H., Z.L., and E.J.B. performed experiments; L.A.J., Z.L., and E.J.B. analyzed data; L.A.J., Z.L., and E.J.B. interpreted results of experiments; L.A.J., L.H., and E.J.B. prepared figures; E.J.B. drafted manuscript; L.A.J., L.H., Z.L., and E.J.B. edited and revised manuscript; L.A.J., L.H., Z.L., and E.J.B. approved final version of manuscript.
